# Surface electromyography using dry polymeric electrodes

**DOI:** 10.1063/5.0148101

**Published:** 2023-09-11

**Authors:** Nicolas Steenbergen, Ivan Busha, Alexis Morgan, Collin Mattathil, Arieh Levy Pinto, Fotios Spyridakos, Ivan Sokolovskiy, Bogachan Tahirbegi, Christopher Chapman, Estelle Cuttaz, Karina Litvinova, Josef Goding, Rylie Green

**Affiliations:** Department of Bioengineering, Imperial College London, London SW7 2BP, United Kingdom

## Abstract

Conventional wet Ag/AgCl electrodes are widely used in electrocardiography, electromyography (EMG), and electroencephalography (EEG) and are considered the gold standard for biopotential measurements. However, these electrodes require substantial skin preparation, are single use, and cannot be used for continuous monitoring (>24 h). For these reasons, dry electrodes are preferable during surface electromyography (sEMG) due to their convenience, durability, and longevity. Dry conductive elastomers (CEs) combine conductivity, flexibility, and stretchability. In this study, CEs combining poly(3,4-ehtylenedioxythiophene):polystyrenesulfonate (PEDOT:PSS) in polyurethane are explored as dry, skin contacting EMG electrodes. This study compares these CE electrodes to commercial wet Ag/AgCl electrodes in five subjects, classifying four movements: open hand, fist, wrist extension, and wrist flexion. Classification accuracy is tested using a backpropagation artificial neural network. The control Ag/AgCl electrodes have a 98.7% classification accuracy, while the dry conductive elastomer electrodes have a classification accuracy of 99.5%. As a conclusion, PEDOT based dry CEs were shown to successfully function as on-skin electrodes for EMG recording, matching the performance of Ag/AgCl electrodes, while addressing the need for minimal skin prep, no gel, and wearable technology.

## I. INTRODUCTION

There is an increasing demand for long-term electroceutical devices in clinical therapeutics, wearable interfaces, and neuroprosthetics. The current gold standard for skin surface biopotential recording applications is wet Ag/AgCl electrodes.^1^ Ag/AgCl electrodes have an electrolyte gel between the electrode and skin surfaces to facilitate ion exchange, increase conductivity, and provide a buffer layer.^1–3^ Consequently, the gel reduces contact impedance and motion artifact, minimizing the noise of the extracted signal. In turn, this yields a high signal-to-noise ratio (SNR).^1–3^ However, wet electrodes have limitations such as extensive skin preparation, dermatological reactions to the gel, restricted longevity due to gel drying, and durability.^1–3^ Commercial wet metallic devices with an expanded number of channels are also unsuitable for long-term use due to poor placement repeatability and reduced signal intensity over time.^1–3^

As an alternative to wet metallic electrodes, dry electrodes are very promising. They do not use an electrolyte gel, and therefore, they are able to be used for longer-term recording.^1^ However, metallic versions of dry electrodes are suboptimal due to their stiffness and resistance to flexion. The resulting mechanical mismatch causes decreased signal intensity, increased contact impedance, motion artifact, and the likelihood of device failure. This increases the noise of the extracted signal compared to wet metallic electrodes and results in a comparatively lower SNR.^1,4^ Multi-channel commercial dry metallic devices are reported to have a 99% classification accuracy but are expensive, bulky, and uncomfortable to compensate for poor interfacing with skin.^5–8^ There is, thus, a need for electrodes with similar function to conventional dry and wet metallic electrodes but able to better interface with the skin and provide a long-term biopotential recording. Conductive elastomer (CE) electrodes present a viable alternative to dry polymeric electroactive composites that provide flexibility, stretchability, and conductivity to produce smaller electrodes with reduced mechanical mismatch and motion artifact.^1,9–12^ O'Brien *et al.*^12^ reported that CEs have substantially larger peaks and root mean square (RMS) measurements than Ag/AgCl electrodes, stating that they would improve current hand prosthetic control systems.

However, there is a wide range of conductive elastomers presented in the literature based on various polymer carrier materials and conductive components. Most dry CE electrodes consist of an elastomeric carrier and a conductive filler.^13^ The conductive filler is typically metallic particles, carbon-based particles, or conjugated polymers.^14^ Metallic particles are favored for their high conductivity, leading to the litany of studies that combine silver or titanium nitride with an elastomeric substrate to produce dry electrodes that perform favorably compared to wet electrodes.^15–24^ However, metallic electrodes run the risk of oxidation or corrosion, and silver has been associated with adverse skin reactions.^25^ Carbon-based components come in different forms, such as carbon, carbon-black, carbon nanotubes, carbon nanofibers, and graphene, each with their distinct advantages and disadvantages. As a result, these materials have formed the basis of a large number of studies in the dry electrode space.^26–34^ Carbon-based conductive fillers generally have good mechanical and electrical properties but are typically difficult to disperse, have controversial safety, or can be expensive.^14^ Conjugated polymers vary more in their behavior but are well-tolerated in contact with the body, having a history of use in implantable applications.^14,35,36^ The most commonly used conjugated polymers are polypyrrole (PPy), polyaniline (PAni), polythiophene (PTh), and its derivative poly(3,4-ethylenedioxythiophene) (PEDOT).^37^ PPy is the most extensively investigated polymer due to a high electrical conductivity and its relative ease in processing, but it is brittle and electrochemically unstable.^38^ Although PAni is cost-effective and environmentally stable, it is difficult to process.^39^ PTh has an excellent conductivity but also falls short where PAni does with processing difficulties.^39^ PEDOT-based compounds, in particular the commercially available PEDOT complexed to the polymeric dopant poly(styrenesulfonate) (PEDOT:PSS) have a slightly lower conductivity in exchange for a high storage capacity, low Young's modulus, and low interface impedance.^35^ For these reasons and their established cytocompatibility, PEDOT is now heavily investigated as the conjugated polymer of choice due to suitable mechanical and electrical properties for biopotential recording applications.^40^

PEDOT:PSS based electrodes are, thus, a promising alternative, and they have been adapted in several studies for electromyography (EMG). Nijima *et al.*^41^ used commercial PEDOT:PSS impregnated textile electrodes to develop an EMG device to monitor mastication muscle activity, reporting high correlation coefficients for RMS and movement. However, this study was performed on only one subject. Zucca *et al.*^42^ used PEDOT:PSS tattoo electrodes to investigate hand movements and reported comparable function to wet Ag/AgCl electrodes, the ability to control a robotic arm with five classified movements, and the use of electrodes for up to 12 h without degradation or dermatological reaction. However, these novel methods using temporary tattoo paper transfer do not allow for repeated use, and experiments were performed on only one subject. Zhang *et al.*^43^ produced flat film electrodes with PEDOT:PSS, WPU, and D-sorbitol (PWS), reporting the ability in EMG tests to distinguish gripping forces, and the flexion and extension of individual fingers. These electrodes were also used in a clinical setting on the biceps to measure the muscular reflex response elicited by a tendon hammer and distinguish muscular exertion during an isometric contraction test at an increasing load. Despite promising results, the number of subjects these electrodes were tested on is unclear, there is no comparison of the PWS electrodes to gold standard Ag/AgCl electrodes, and there is no evidence of electrode reusability, giving little context of the improvement of these electrodes upon the current standard. Recently, Tan *et al.*^44^ produced a composite PEDOT:PSS and supramolecular solvent polymer that has self-adhesive properties and trades a slight drop in conductivity for significantly enhanced mechanical properties. They also tested this electrode in an EMG setting to detect biopotentials at different gripping forces at a comparable performance to commercial Ag/AgCl electrodes. However, it is unclear how accurate the grip strength classification ability is, as these data are not presented.

It is apparent that the elastomeric carriers that these conductive fillers are in contributed to the overall electrode function. The PEDOT:PSS is typically placed on or in thermoplastic or waterborne polyurethanes (TPU or WPU), polydimethylsiloxane (PDMS), foams, silicone, or integrated into various textiles to produce recording devices.^45^ Waterborne polyurethanes are known for their eco-friendliness and processing ease, but their implementation in dry electrodes sacrifices water and solvent resistance, thermal stability, and mechanical strength, which are vital for biopotential electrodes.^46^ Thermoplastic polyurethanes are suitable for a large range of fabrication techniques and are primarily known for being mechanically resilient and flexible, making them favored for medical applications where these properties are integral to long-term function.^47^ PDMS and other silicones are used in existing medical devices due to their cytocompatibility, chemical inactivity, thermal stability, and water and oxidation resistance.^46^ Conjugated polymer foams and textiles are a more recent area of research aiming to produce flexible, moisture permeable, and easily integrable soft electrodes for wearable medical devices.^14^ Cuttaz *et al.*^36^ produced solid PEDOT:PSS dispersed in thermoplastic polyurethane (PU) electrodes for an implantable context, reporting a conductivity of 7.13 ± 0.44 S cm^−1^ at 20 wt. % and a range of properties related to an implant environment. These, thus, differ from other high-functioning elastomeric electrodes in the literature as they use thermoplastic polyurethane as opposed to the waterborne polyurethane electrodes produced by Zhang *et al.*^43^ The electrodes of Cuttaz *et al.* are already well-characterized and have been reported on several times for implantation studies.^36,48^ However, their ability to measure surface EMG (sEMG) as a solid reusable electrode has not been investigated. This study's principal aim is to provisionally determine if these implantable electrodes can also be applied to EMG.

So, this study focused on adapting 25 wt. % PEDOT:PSS/thermoplastic PU CE electrodes developed for implant devices to an sEMG setting.^36^ It was hypothesized that the PEDOT:PSS/PU CE electrodes can maintain adequate contact and exhibit a low impedance due to its capacity for ionic charge transfer at low frequencies, enabling a high quality dry recording signal.

**FIG. 1.**
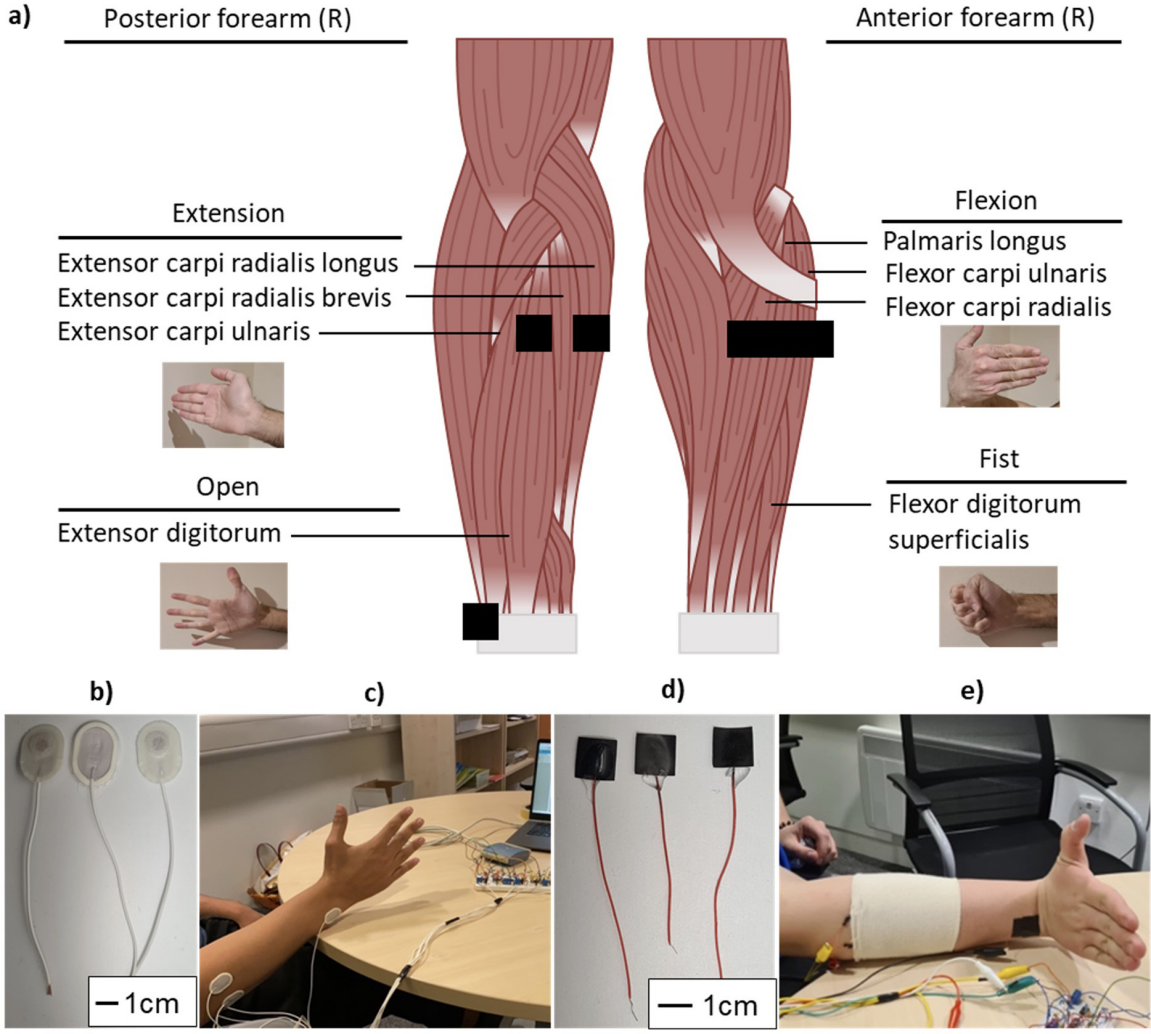
Anatomical diagram of the anterior and posterior muscular compartments of the forearm showing the electrode placement locations and corresponding muscles and movements (a), used wet Ag/AgCl electrodes (b) on the forearm (c) and dry solid PEDOT:PSS electrodes (d) on the forearm (e).

To interrogate this hypothesis, the aim was to compare the properties of wet Ag/AgCl and dry CE electrodes as skin contacting electrodes. This was achieved via electrochemical testing, functional EMG testing on the forearms of four subjects, and classification testing of the acquired EMG signals. For this, six electrodes were placed on forearm muscles corresponding to pre-determined movements [Fig. 1(a)].^49–51^ A reference electrode was placed on the ulnar prominence at an electrically inert location.^2,49^ The same six CE electrodes and CE reference electrode were used for all subjects, whereas all Ag/AgCl electrodes were replaced for every subject. A circumferential configuration was applied to reflect prior literature studies in forearm sEMG and emulate future practical use of the electrodes as a wearable band.^8,51,52^ Wet Ag/AgCl sEMG electrodes [Figs. 1(b) and 1(c)] and solid dry PEDOT:PSS/PU [Figs. 1(d) and 1(e)] electrodes were directly compared on each subject.

CE electrodes were made into 1 cm squares, matching the active electrode area of commercial wet Ag/AgCl sEMG electrodes and the maximum recommended area per SENIAM guidelines.^50^ Four participants were used in line with relevant major studies.^6,41,42^ Each movement was performed 30 times for 3 s across five trials, totaling 150 contractions per movement and 600 contractions overall per participant.

From a group of such movements, many studies determine the classification accuracy to compare electrodes as it is a realistic anticipation of the future application of the electrodes.^5,42^ The resulting classification accuracy is highly dependent on the system at hand, but an accuracy close to that of the gold standard test is an indicator of electrode functionality in this real-world setting.^5,42,53,54^ To determine classification accuracy, the sEMG used a backpropagation artificial neural network (BPANN), which has been found to be an effective tool for real-time EMG signal classification.^55^ BPANN is a method that estimates the total loss due to each node and inserts it back into the neural network. Then it minimizes the loss by giving nodes with higher error rates lower weights and re-estimating loss until optimal node weights are achieved. This approach has been used in previous studies with high accuracy.^56^ In this investigation, it is found that CE electrodes were functionally comparable to Ag/AgCl electrodes.

## II. RESULTS AND DISCUSSION

### A. Material characterization

**FIG. 2.**
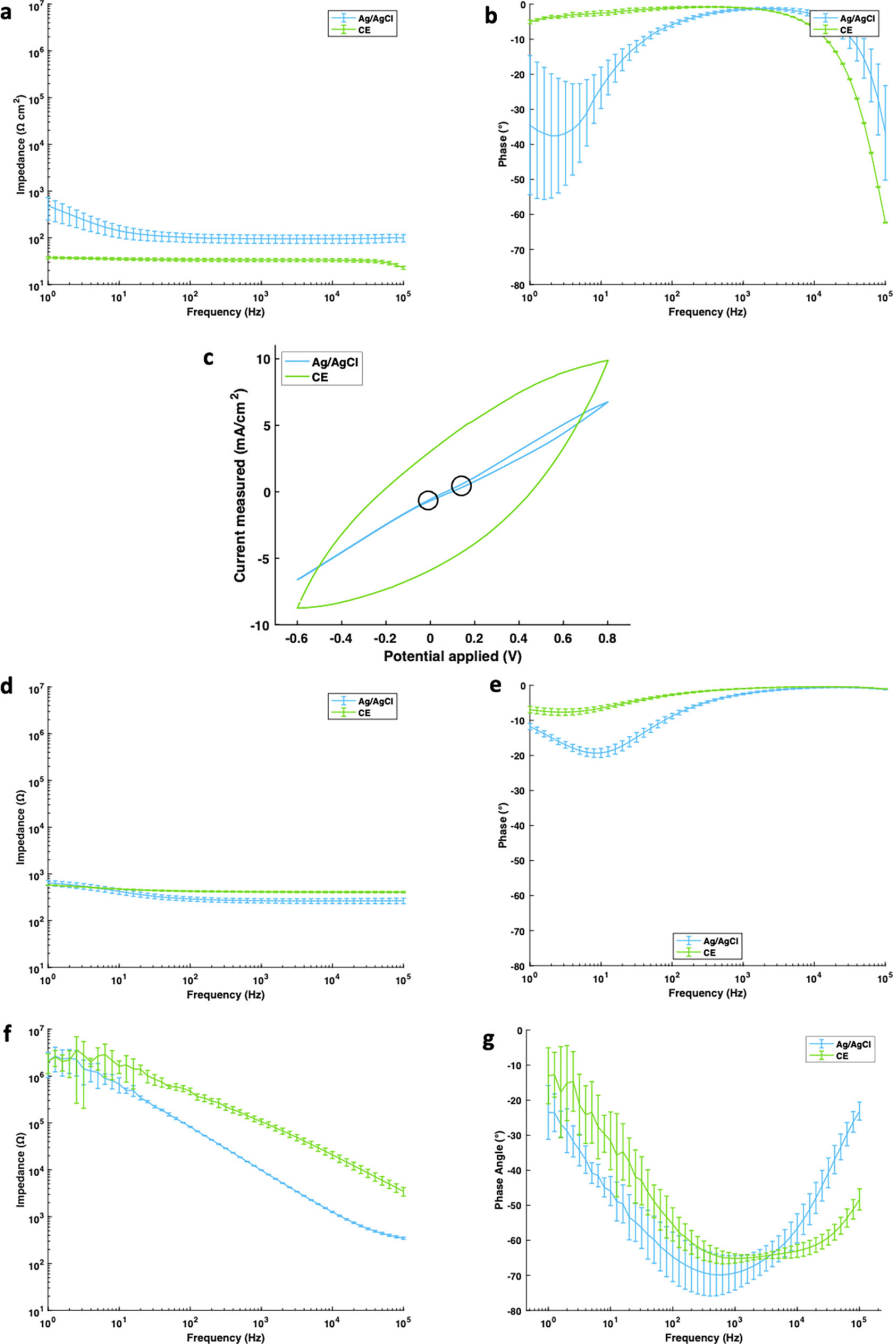
Graphs showing (a) wet impedance, (b) wet phase angle, (c) wet cyclic voltammetry, (d) skin phantom impedance, (e) skin phantom phase angle, (f) live skin impedance, and (g) live skin phase angle. Data show the mean ± standard deviation (n = 3).

The CE electrode mechanical properties and surface morphologies have already been investigated by Cuttaz *et al.*^36,48^ Electrochemical performance of the CE and Ag/AgCl electrodes (n = 4) was investigated in a standard wet cell environment via electrochemical impedance spectroscopy (EIS) and cyclic voltammetry (CV). CV and EIS aim to understand the fundamental charge transfer mechanism of both the CE and Ag/AgCl electrodes and show that the CE electrochemical properties significantly differ from those of the Ag/AgCl electrodes. The Ag/AgCl impedance spectrum [Fig. 2(a)] increases at low frequencies whereas the CE response is more stable and resistive, showing a frequency-independent impedance profile. It is important to note that this is a wet cell environment, where the PBS can penetrate straight to the electrode surface, and hence, the capacitive transfer process may dominate over the Faradaic Ag/AgCl reaction. The Ag/AgCl electrodes, thus, show a capacitive profile, in line with the literature, due to the electric double layer forming at the Ag surface.^57^ This behavior occurs at low frequencies, suggesting this may be due to slower ion transfer in the conductive gel at this point. As a result, the CE electrodes have lower impedance, particularly at low frequencies. At 1 kHz, the CE has an impedance of 51.3 ± 13.2 Ω cm^2^. This value is two magnitudes smaller than the Ag/AgCl electrodes at low frequencies. This is significant because the useful frequency ranges of most bioelectric signals are typically below this, i.e., EEG: 0–50 Hz, electrocardiography (ECG): 50–100 Hz, EMG: 50–150 Hz, although EMG signals can be measured up to 10 kHz.^13,45,58^ Additionally, having a lower impedance in this range also translates into lower noise levels. CE electrodes are, thus, better adapted to meaningful biopotential measurement than Ag/AgCl electrodes.

Furthermore, the phase spectra and CV of the materials are critical to understand the charge transfer behavior of the electrodes [Fig. 2(b)]. The Ag/AgCl electrode experiences a ≈−30° change around 10 Hz, in line with the impedance change spectrum. This again indicates the presence of some capacitive behavior, but not complete, as a true capacitor has a phase lag of −80° or −90°. The nearly flat line of the CE electrodes indicates there is little capacitive behavior, which is typical of PEDOT and other conductive polymer electrodes.

The CV measurements further show the difference between CE and Ag/AgCl electrochemical behavior. Ag/AgCl electrodes show two small points of inflection [Fig. 2(c)] indicative of the reduction and oxidation reactions as part of its charge transfer process. CEs do not have any distinguishable peaks, suggesting that the peaks associated with the redox chemistry are smaller. CEs have a considerably larger hysteresis curve when compared to Ag/AgCl, showing their ability to store charge. This characteristic allows for the implementation of CE electrodes in closed-loop systems and stimulation circuits by virtue of being able to provide a feedback signal.

**FIG. 3.**
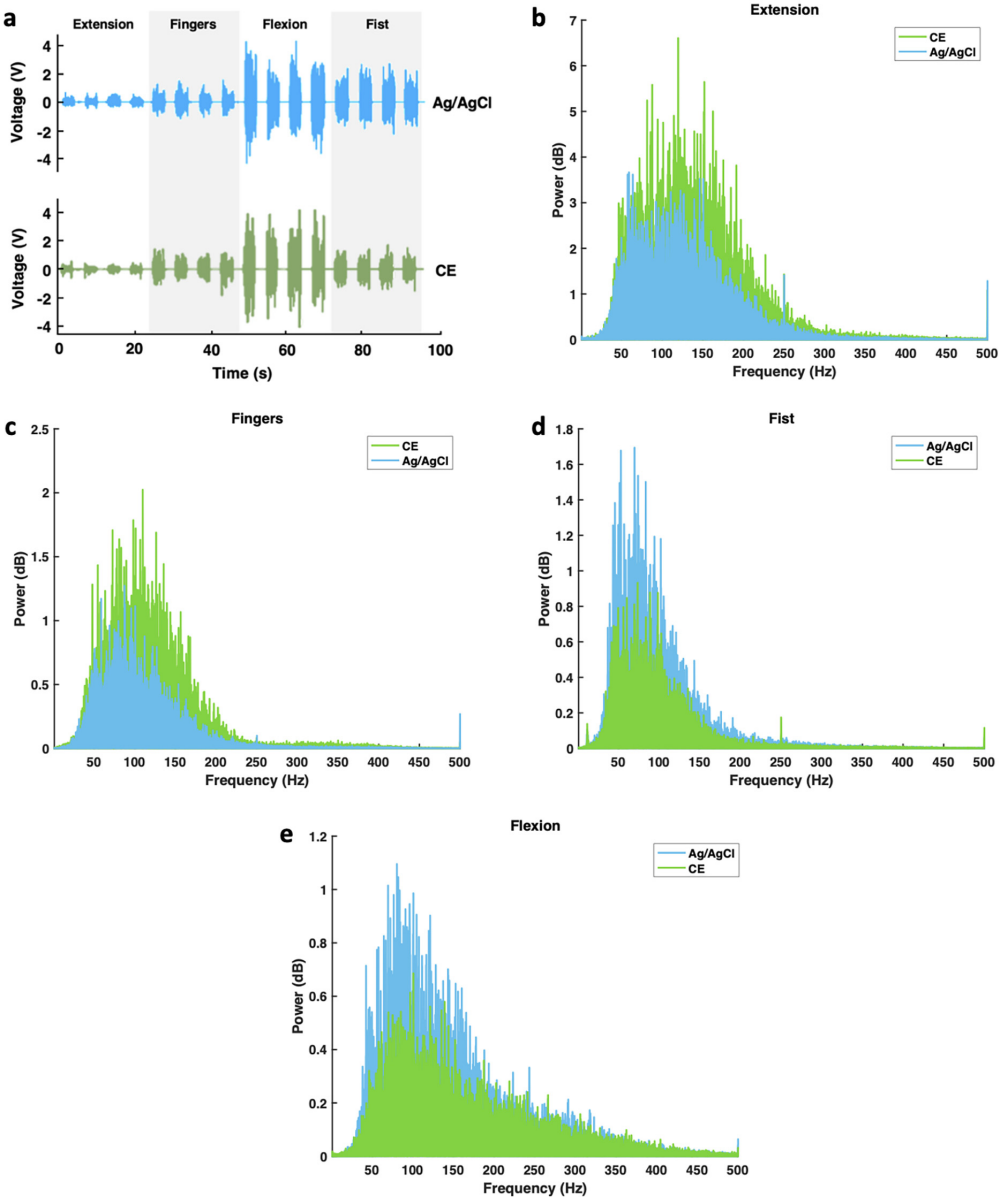
Assessment of Ag/AgCl and CE electrodes via (a) time-domain EMG recordings acquired during the “rest” and “onset of muscle contractions” conditions and average power spectra during (b) extension, (c) open, (d) fist, and (e) flexion movements.

Further EIS was performed on a phantom skin model, showing similar behavior in the CE electrodes [Figs. 2(d) and 3(e)]. Despite a lesser degree, the Ag/AgCl electrodes also showed some capacitive behavior indicated by the −6° drop in the phase angle accompanied by a 300 Ω increase in impedance. Finally, EIS was also performed on human skin [Figs. 2(f) and 2(g)]. Here, the behavior of both electrodes changed drastically, but this is not often disclosed or discussed in other studies. Both the CE and Ag/AgCl electrodes exhibited capacitive behavior with a −15° and −55° change in the phase angle, accompanied by a matching increase in impedance to around 200 kΩ. This capacitive behavior is most likely explained by the capacitive nature of the skin, since this is the only factor that changed. All findings are in line with the previous literature.^14,36,59^

### B. Signal quality

Electrodes were placed circumferentially on forearm muscles to record pre-determined movements. Specifically, wrist extension predominantly recruits the carpi radialis longus, carpi radialis brevis, and carpi ulnaris extensor muscles. The electrodes on top of these muscles dominate the EMG signal during extension whereas the anterior forearm electrodes receive a reduced signal. In contrast, flexion primarily recruits the palmaris longus and carpi ulnaris and carpi radialis flexor muscles. Thus, these electrodes on top of these muscles dominate during flexion. These two movements are, therefore, easily visually distinguishable. The fist movement recruits flexor digitorum superficialis muscle, which activates one or two of the anterior forearm electrodes. This makes it like the flexion EMG signal but still distinguishable based on the activated electrodes. The open movement primarily recruits the extensor digitorum, which is a smaller muscle and will, thus, produce a smaller EMG signal compared to extension, making it distinguishable in the posterior forearm electrodes.^51^ Although all electrodes will measure EMG signals in all movements, the predominant muscle recruitment produces different EMG patterns that can then allow for visual verification.

The extracted signals [Fig. 3(a)] were qualitatively similar between the Ag/AgCl and CE electrodes, although the Ag/AgCl electrodes appear to have higher peaks in some instances. This is corroborated by the power spectra of both electrodes showing similar shape and power [Fig. 3(b)].

**FIG. 4.**
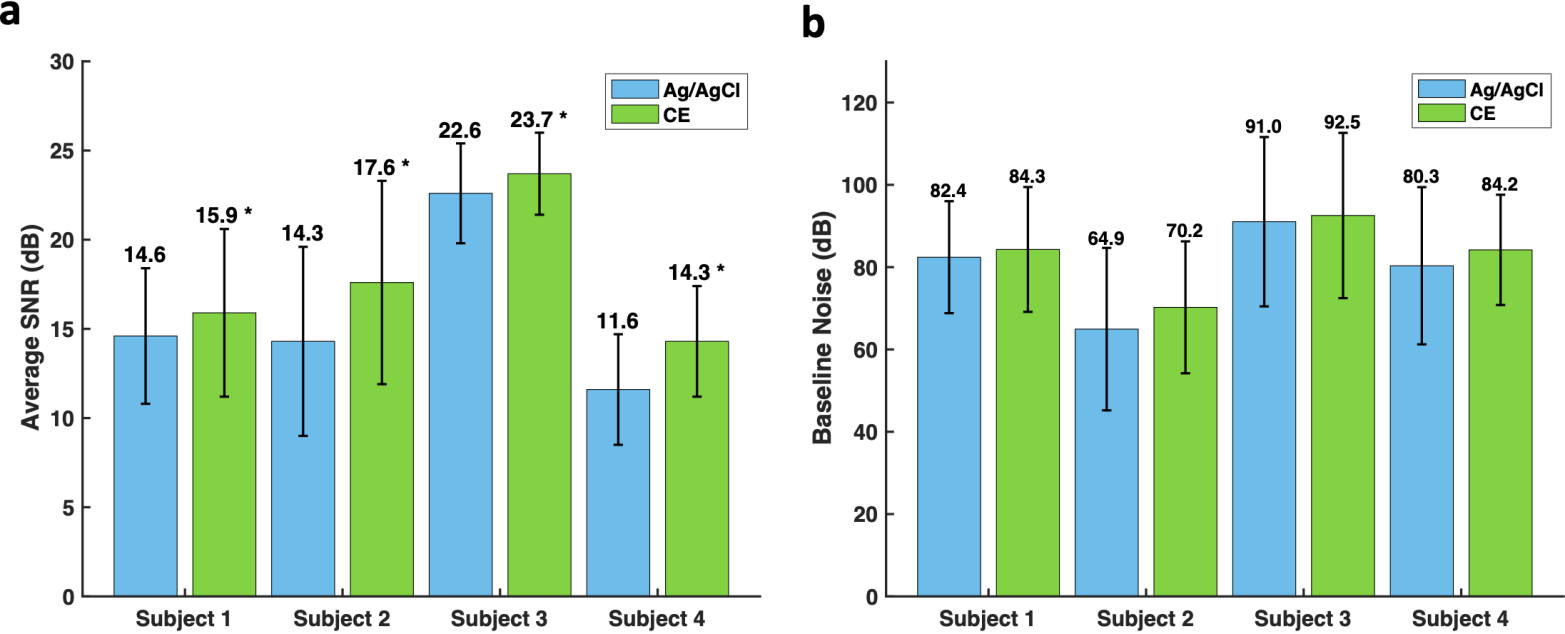
Bar charts of (a) average SNR and (b) baseline noise per subject for Ag/AgCl and CE electrodes.

The same six CE electrodes were used for all movements, trials, and participants, whereas the six Ag/AgCl electrodes were replaced between every participant in approximately 30-min intervals to prevent gel drying and the associated drift and artifact. The overall signal-to-noise ratio (SNR) of commercial Ag/AgCl electrodes was 15.7 ± 0.3 dB and of CE electrodes was 18.2 ± 0.3 dB (p < 0.0001). Despite the inter-subject variability displayed in the SNR, CE electrodes consistently maintained a significantly higher SNR than Ag/AgCl electrodes in all subjects (p < 0.0001) [Fig. 4(a)]. Both the overall recorded SNR of the Ag/AgCl and CE electrodes are within the reported ranges of 11–56 and 10–24 dB, respectively.^60–64^

Additionally, the baseline noise of the CE electrodes remained similar to the baseline noise of the Ag/AgCl electrodes across all subjects [Fig. 4(b)] and, thus, the entire recording period. However, comparable baseline noise combined with the higher SNR in CE electrodes indicates that CE electrodes could maintain a higher quality signal for at least 2 h, in line with the previous literature.^12^ This also indicates that the electrodes could potentially be used as reusable electrodes as they maintain a similar or superior signal than the single use Ag/AgCl electrodes. However, this investigation was not designed to test reusability of the CE electrodes, and so this must be tested further.

### C. Feature extraction and BPANN performance

**FIG. 5.**
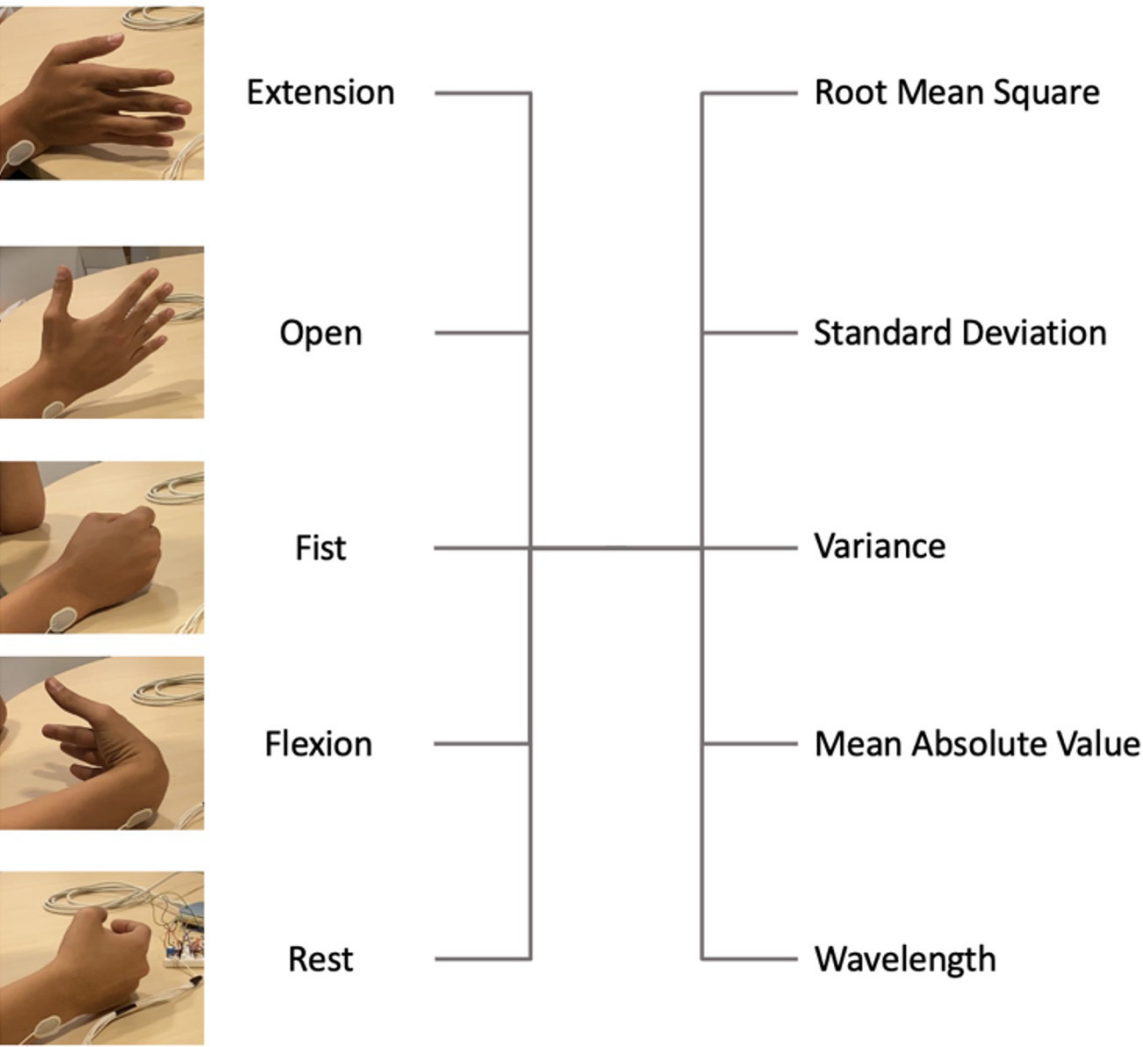
A schematic of the movements and the extracted features to accompany Table I.

To perform classification, the neural network requires inputs. The features extracted from the signal used as inputs are root mean square (RMS), standard deviation (SD), variance (VAR), mean absolute value (MAV), and wavelength (WL) (Fig. 5 and Table I). These show high accuracy in combination with a BPANN in the literature.^65–67^ Table I shows the features per movement per electrode averaged over all participants for channel 1.

**TABLE I.** Table showing the values of all the extracted features averaged over all participants (n = 4) for channel 1 per electrode per movement. Data show the mean ± standard deviation.

Movement	Electrode	RMS	SD	VAR	MAV	WL
Extension	Ag/AgCl	0.044 ± 0.058	0.043 ± 0.058	0.005 ± 0.064	0.022 ± 0.022	44.4 ± 66.1
	CE	0.030 ± 0.045	0.029 ± 0.045	0.003 ± 0.034	0.014 ± 0.011	27.9 ± 23.4
Open	Ag/AgCl	0.079 ± 0.058	0.078 ± 0.059	0.010 ± 0.012	0.043 ± 0.032	103.2 ± 87.4
	CE	0.065 ± 0.039	0.064 ± 0.040	0.006 ± 0.006	0.034 ± 0.019	86.5 ± 63.1
Fist	Ag/AgCl	0.149 ± 0.090	0.149 ± 0.091	0.030 ± 0.043	0.088 ± 0.054	182.0 ± 148.9
	CE	0.117 ± 0.054	0.117 ± 0.054	0.017 ± 0.015	0.068 ± 0.033	139.3 ± 81.6
Flexion	Ag/AgCl	0.259 ± 0.124	0.258 ± 0.124	0.082 ± 0.075	0.153 ± 0.075	449.9 ± 290.5
	CE	0.223 ± 0.100	0.223 ± 0.100	0.060 ± 0.068	0.125 ± 0.053	402.9 ± 223.6
Rest	Ag/AgCl	0.023 ± 0.057	0.020 ± 0.057	0.004 ± 0.085	0.010 ± 0.025	9.9 ± 67.6
	CE	0.014 ± 0.057	0.012 ± 0.024	0.001 ± 0.004	0.007 ± 0.012	7.0 ± 31.5

The extracted data were used to train a Ag/AgCl BPANN and a PEDOT:PSS/PU BPANN and optimized by tuning multiple hyperparameters (Table II). The Ag/AgCl and PEDOT:PSS/PU networks have learning rates of 0.01 and 0.05, respectively. All networks have 20 first-order hidden neurons and eight second-order hidden neurons. The batch sizes are 256 and 128, respectively. The stopping epochs are 542 and 180, respectively. No networks had a dropout.

**TABLE II.** Table showing the hyperparameters and training, validation, and testing accuracies of the Ag/AgCl trained and PEDOT:PSS trained BPANNs.

Hyperparameters	Ag/AgCl BPANN	PEDOT:PSS BPANN
Features	RMS, MAV, VAR, WL, SD	RMS, MAV, VAR
Learning rate	0.01	0.05
Nodes	(20, 8)	(20, 8)
Batch size	256	128
Dropout	No	No
Stopping epoch	542	180
Training accuracy	99.49%	99.16%
Validation accuracy	99.13%	99.13%
Testing accuracy	98.47%	99.57%

**FIG. 6.**
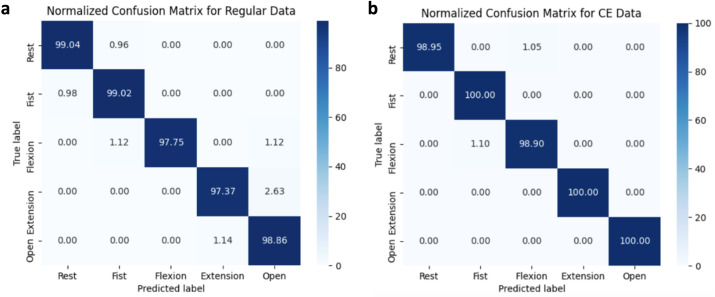
Confusion matrices of (a) Ag/AgCl EMG data tested on an Ag/AgCl trained BPANN and (b) CE EMG data tested on a CE trained BPANN.

The Ag/AgCl neural network achieves its highest testing accuracy of 98.47% with analysis of all extracted features. Its training and validation accuracies are 99.49% and 99.13%, respectively (Fig. 6). The PEDOT:PSS/PU neural network achieves its highest testing accuracy of 99.57% with analysis of RMS, VAR, and MAV. Its training and validation accuracies are 99.16% and 99.13%, respectively. The data were also cross-tested to determine the translatability of the extracted electrode data. Ag/AgCl data tested on the PEDOT:PSS/PU trained BPANN achieve a classification accuracy of 85.98%, whereas PEDOT:PSS/PU data tested on the Ag/AgCl trained BPANN achieves a classification accuracy of 94.30%.

Previous studies using PEDOT:PSS tattoo electrodes achieved an average classification accuracy of at least 90% with a support vector machine (SVM) neural network.^42^ Studies using different CE electrodes achieved classification accuracies of 84%–99%, while achieving similar accuracies with Ag/AgCl electrodes.^5–8,11^ Thus, our electrodes and neural network perform similarly to existing studies, but it is difficult to directly compare these results due to the use of different neural networks and combinations of extracted features. Despite this, CE electrodes have previously shown good classification accuracies when compared to Ag/AgCl electrodes in hand gesture experiments.^51,54,55,65,68–70^ A significant finding not previously investigated is that CE electrode data on an Ag/AgCl trained BPANN still have a high classification accuracy, indicating that CE electrodes can be used with existing gesture recognition systems. However, more rigorous testing with more subjects is required to confirm this observation.

Current gesture recognition systems have several uses, including wheelchair control, robotic hand control, prosthesis control, rehabilitative stimulation, and computer interface control by placing electrodes on hands, arms, legs, the face, and inside prostheses. Classification accuracies of Ag/AgCl and CE electrodes in these devices are typically 80%–100%.^5,42,52,54,66,69,71^ However, the use of dry Ag/AgCl electrodes produces bulky commercial devices that can be streamlined with CE electrodes, since these show a similar or superior performance. Thinner more streamlined systems can also accommodate force myography sensors, which have been used to improve the classification of a greater number of gestures. McIntosh *et al.*^52^ achieved a classification of 96% with this setup, while Jiang *et al.*^54^ achieved a classification accuracy of 96.7% with 48 hand gestures. More importantly, the potential for translatability means that it could be possible to easily swap existing commercial Ag/AgCl electrodes with new CE electrodes while still maintaining a high classification accuracy.

A known problem of dry electrodes is that a minimum baseline adhesion pressure is required to extract a viable signal.^43^ This problem is not present in tattoo electrodes because the tattoo film maintains a high adhesion pressure. While an elastic armband was used in this investigation to ensure sufficient contact with the skin, this also results in a bulkier device. Therefore, future studies will focus on the integration of dry CE electrodes into a wearable armband.

Previous studies have attempted to address this issue in two major ways: patterning and adhesive substrates. Patterning of microfeatures on a PEDOT:PSS electrode surface has taken on several shapes over the years. Penetrating microneedles, micropillars, suction cups, gecko feet, and topographical patterning have all been used to attempt to improve adhesion and connection with the skin, particularly when it has significant hair growth.^14,43,71–74^ The use of polydimethylsiloxane (PDMS), cellulose layering, and D-sorbitol have also shown increased adhesive properties.^42,43,75–80^ There are, thus, a wide range of methods to use, besides tattoo film, to improve the adhesion of electrodes to the skin while maintaining reusability.

## III. CONCLUSION

In this proof-of-concept study, solid reusable CE electrodes exhibit the ability to produce EMG data similar or superior to gold standard wet Ag/AgCl electrodes. The CE displayed a significantly higher SNR (18.2 ± 0.3 dB) than the wet Ag/AgCl electrodes (15.7 ± 0.3 dB) (p < 0.0001). Both electrodes portrayed a stable signal throughout the experiment, while CE electrodes maintained a consistently lower signal drift, showing the ability of CE electrodes to be reused. However, the limit of reusability was not determined. Furthermore, the CE electrodes achieved a high classification accuracy of 99.57% on a CE-trained BPANN and 94.30% on an Ag/AgCl-trained BPANN. This indicates that solid CE electrodes can be used and implemented in existing movement classification frameworks.

## IV. METHODS

### A. Participants

Five males volunteered for this study. Exclusion criteria included neurological conditions affecting muscle and nerve conduction. One subject was excluded due to a severed ulnar nerve. Thus, four healthy males (20 ± 2 yr) were included in this study.

### B. Materials

Conventional wet Ag/AgCl electrodes (Neuroline 720 surface electrodes) are purchased from Ambu. Unless stated otherwise, all reagents were purchased from Sigma Aldrich. Thermoplastic PU elastomer pellets (Pellethane 2363–80AE Polyurethane Elastomer, Ether based) were purchased from Velox GmbH. All circuit components were purchased from RS components, and the ADC (USB-1208FS-plus) was purchased from Measurement Computing. The armband used to secure CE electrodes was a size B tubular support bandage from Boots Pharmaceuticals.

### C. Fabrication of conductive elastomers

PU films were solvent cast from dimethylacetamide (DMAC) solutions containing 5 wt. % (w/v) PU. PU was dissolved in DMAC at 60 °C for 24 h prior to the addition of 0.16 wt. % (w/v) of lithium perchlorate (LiClO_4_). PEDOT:PSS was dispersed in the PU solution (25 wt. %) by stirring for 3 days at 60 °C. PEDOT:PSS/PU solutions were cast onto glass plates in a vacuum oven (BINDER GmbH) at an initial isotherm of 60 °C for 30 h.

### D. Electrode production

CE electrodes were laser cut into 1 cm^2^ squares with a Lotus Meta-c laser system. Resultant electrodes were bonded to wires and insulated with silicone. The entire production process is modified from a previous study.^13^ A total of 12 electrodes were produced.

### E. Electrochemical characterization

Electrochemical characterization of CE films and Ag/AgCl electrodes comprised electrochemical impedance spectroscopy (EIS) and cyclic voltammetry (CV). Wet EIS and CV were conducted under ambient atmospheric conditions employing a conventional three-electrode cell, equipped with a platinum (Pt) counter electrode and an isolated Ag/AgCl reference electrode. Phosphate buffered saline (PBS) was used as the electrolyte. EIS was investigated by the application of a 10 mV sinusoidal voltage between the working and reference electrodes across the frequency range of 0.1 Hz–10 kHz. EIS on the skin phantom and human skin was conducted using a Spes Medica 35 × 45 mm^2^ disposable adhesive surface electrode as the counter/reference electrode. The skin phantom was composed of a 30 g/l agarose gel with a 2 g/l NaCl concentration. On human skin testing, the working electrode was placed on the ulnar prominence, and the counter electrode was placed on the opisthenar area. CV was evaluated by sweeping the voltage between −0.6 and 0.8 V at a 0.15 V s^−1^ scan rate, and the current response was measured. All the measurements were accomplished with an AUTOLAB potentiostat–galvanostat (Multi Autolab/M101, Eco Chemie, Netherlands) and the corresponding software Nova.

### F. Circuit production

A circuit board was independently produced, placing electrodes in four channels via a bipolar configuration. This allows the signal common to both electrodes, such as noise, to be attenuated and differences to be amplified with a gain of 100.^2,81,82^ A 700 Hz active low pass filter is implemented to prevent aliasing. A non-inverting amplifier is used with a gain of 34 to provide sufficient amplification and is connected to an Analog-to-Digital Converter at 2000 S/s, in line with the previous literature.^68,82,83^

### G. Experimental protocol

Before testing, each participant's dominant forearm (all right-handed) was shaved and cleaned with isopropyl alcohol.^2^ Electrodes were applied to the skin according to Fig. 2. An armband was applied over the PEDOT:PSS electrodes. Including preparation, each electrode is used up to 2.5 h.

For each movement, participants performed the following protocol:

(1) 6 s of rest to establish baseline activity,
(2) 30 cycles of a 3 s maximal voluntary contraction (MVC) followed by 3 s of rest,
(3) this is repeated for four more trials to a total of five trials, resulting in an overall 150 contractions per movement and 600 contractions per participant, and
(4) the first four trials are used to train the neural networks, while the last trial is used to test the neural networks.

### H. Digital signal filtering

The input csv file has a binary column renamed “movement,” which takes a value of 1 during a MVC and a value of 0 during a rest period. Due to the recording protocol of repeating periods of 3 s of MVC and 3 s of rest, the movement column is a repeating pattern of 0 s followed by 1 s until the end of the recording. Acquired signals are processed via wavelet transform.^53,84^ Optimization shows the Haar wavelet (db1) to be most effective for this data sample. Each contraction and rest period are manually spliced and sorted into a corresponding file. Splicing location and length are adjusted for every subject according to their signal intensity and frequency. Offset is also digitally removed to normalize all signals.

### I. Feature extraction

Features are extracted for each MVC and rest period. A produced algorithm (https://github.com/ariehlev/Group-Project-Code) parses the movement column and saves the indices when there is a change in this column (from both 0 to 1 or from 1 to 0). This makes it possible to extract features for each MVC and resting period. The features extracted are RMS (root mean square), mean absolute value (MAV), variance (VAR), standard deviation (SD), and waveform length (WL). The equations are as follows:^85–87^

$$\mathrm{RMS}=\sqrt{\frac{1}{N}\sum_{n=1}^{N}{\left|x_{n}\right|}^{2}},$$ (1)

$$SD=\sqrt{\frac{1}{N-1}\sum_{n=1}^{N}x_{n}^{2}},$$ (2)

$$\mathrm{VAR}=\frac{1}{N-1}\sum_{n=1}^{N}x_{n}^{2},$$ (3)

$$\mathrm{MAV}=\frac{1}{N}\sum_{n=1}^{N}\left|x_{n}\right|,$$ (4)

$$WL=\sum_{n=1}^{N-1}\left|x_{n+1}-x_{n}\right|.$$ (5)

The signal-to-noise ratio for each subject was calculated by first taking a power average of all contraction segments and a power average of all rest segments, which are indicative of the signal and noise portions of the recording, respectively. The average contraction power was then divided by the average rest power to yield the signal to noise ratio.

### J. Classification

**FIG. 7.**
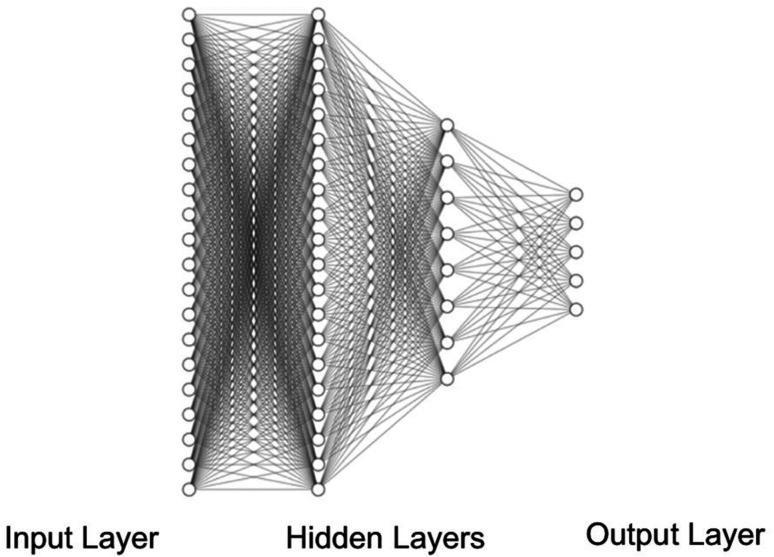
The final structure of both BPANNs with 20 first-order hidden neurons, eight second-order hidden neurons, and five output nodes. Input nodes range from 4 to 20.

Independently produced BPANNs are used in this study, which have shown high accuracies (80%–99%) in previous studies.^65–67,69,88^ The neural network is optimized via careful selection of hyperparameters and features. Two different neural networks are created: one trained on Ag/AgCl electrode data, one trained on PEDOT:PSS-PU data (Table II). The final structure of both neural networks has 20 first-order hidden neurons, eight second-order hidden neurons, and five output nodes (Fig. 7).

## Data Availability

The raw data associated with the human subjects required cannot be shared at this time due to ethical reasons. The processed data and data associated with non-human subjects are available on request.
